# Cerebrospinal fluid characteristics of patients presenting for evaluation of pediatric acute-neuropsychiatric syndrome

**DOI:** 10.3389/fnbeh.2024.1342486

**Published:** 2024-08-19

**Authors:** Rajdeep Pooni, Wynne Zheng, Meiqian Ma, Melissa Silverman, Yuhuan Xie, Bahare Farhadian, Margo Thienemann, Elizabeth Mellins, Jennifer Frankovich

**Affiliations:** ^1^Division of Allergy, Immunology and Rheumatology, Department of Pediatrics, School of Medicine, Stanford University, Palo Alto, CA, United States; ^2^Stanford Immune Behavioral Health Clinic and Research Program, Palo Alto, CA, United States; ^3^Division of Child and Adolescent Psychiatry, Department of Psychiatry and Behavioral Sciences, School of Medicine, Stanford University, Palo Alto, CA, United States

**Keywords:** Pediatric Acute-onset Neuropsychiatric Syndrome (PANS), Pediatric Autoimmune Neuropsychiatric Disorder Associated with Streptococcal Infections (PANDAS), blood brain barrier, obsessive-compulsive disorder, neuroimmune, autoimmune encephalitis

## Abstract

**Objectives:**

This study characterizes cerebral spinal fluid (CSF) indices including total protein, the albumin quotient, IgG index and oligoclonal bands in patients followed at a single center for pediatric acute-neuropsychiatric syndrome (PANS) and other psychiatric/behavioral deteriorations.

**Methods:**

In a retrospective chart review of 471 consecutive subjects evaluated for PANS at a single center, navigational keyword search of the electronic medical record was used to identify patients who underwent lumbar puncture (LP) as part of the evaluation of a severe or atypical psychiatric deterioration. Psychiatric symptom data was ascertained from parent questionnaires and clinical psychiatric evaluations. Inclusion criteria required that subjects presented with psychiatric deterioration at the time of first clinical visit and had a lumbar puncture completed as part of their evaluation. Subjects were categorized into three subgroups based on diagnosis: PANS (acute-onset of severe obsessive compulsive disorder (OCD) and/or eating restriction plus two other neuropsychiatric symptoms), autoimmune encephalitis (AE), and “other neuropsychiatric deterioration” (subacute onset of severe OCD, eating restriction, behavioral regression, psychosis, etc; not meeting criteria for PANS or AE).

**Results:**

71/471 (15.0 %) of patients underwent LP. At least one CSF abnormality was seen in 29% of patients with PANS, 45% of patients with “other neuropsychiatric deterioration”, and 40% of patients who met criteria for autoimmune encephalitis. The most common findings included elevated CSF protein and/or albumin quotient. Elevated IgG index and IgG oligoclonal bands were rare in all three groups.

**Conclusion:**

Elevation of CSF protein and albumin quotient were found in pediatric patients undergoing LP for evaluation of severe psychiatric deteriorations (PANS, AE, and other neuropsychiatric deteriorations). Further studies are warranted to investigate blood brain barrier integrity at the onset of the neuropsychiatric deterioration and explore inflammatory mechanisms.

## Introduction

Changes in cerebrospinal fluid (CSF) may provide important clues to blood brain barrier (BBB) integrity and signs of inflammation. However, for individual patients, the standard CSF evaluation is thought to be insensitive to the diagnosis of inflammatory brain disease (Dale et al., 2017). For example, in a study of CSF characteristics in pediatric-onset multiple sclerosis, 87% of children with early-onset multiple sclerosis had a normal albumin quotient (CSF albumin/serum albumin level) and relatively broad range of total CSF protein (100–720 mg/L) (Pohl et al., 2004). Similarly, normal values have been reported in adult multiple sclerosis with an elevated albumin quotient in only 12–23% of cases (LeVine, 2016). In neuropsychiatric systemic lupus erythematosus (SLE) and autoimmune encephalitis (AE), normal studies do not rule out disease, although CSF total protein, albumin quotient, and IgG index can be abnormal and aid in diagnosis (Stock et al., 2013; Blinder and Lewerenz, 2019). However, in other pediatric inflammatory brain conditions, including acute disseminated encephalomyelitis (ADEM), studies suggest that abnormal CSF studies may be diagnostically valuable to distinguish these diagnoses from others, including multiple sclerosis (Hynson et al., 2001; Lee, 2011; Dale et al., 2017).

There are multiple hypothesized mechanisms involved in post-infectious and autoimmune conditions associated with psychiatric deteriorations (Joubert and Dalmau, 2019). Changes in the permeability of the blood brain barrier is a potential mechanism and is likely involved in lupus, long-COVID, and multiple sclerosis (Gulati et al., 2017; Schreiner et al., 2022; Reiss et al., 2023). An intact BBB limits passage of peripheral mediators of autoimmunity and inflammation, including cytokines and autoantibodies. With persistent exposure to high levels of inflammation, there may be disruption via vascular channels resulting in changes in solute permeability, signaling, cellular traffic and direct damage (Galea, 2021). Abnormalities of CSF total protein and albumin quotient may reflect changes in permeability of the BBB; however, these tests are insensitive if the BBB disruption is limited to a small area (i.e., localized inflammation) (Kadry et al., 2020; Galea, 2021). Multiple methods to quantify BBB permeability *in vitro* have been proposed, however clinical measures are often restricted to proxy measurements of a patient’s serum, cerebral spinal fluid and imaging (Sun et al., 2021). Nonetheless, when studying groups of patients, and more specifically, rare pediatric conditions, understanding the prevalence of CSF abnormalities may help guide next steps in research. This is the goal of this retrospective, descriptive study of CSF characteristics in pediatric subjects with known neuropsychiatric deterioration.

Clinically available but indirect and presumably insensitive measures of BBB disruption include measurements of the albumin quotient and total protein in the CSF. The albumin quotient reflects the altered fraction of CSF versus serum albumin levels and a higher fraction suggests disruption of the BBB (Akaishi et al., 2015). Although fewer than a quarter of patients with pediatric-onset MS have been reported to have an elevated albumin quotient (Pohl et al., 2004), this finding has had predictive significance for increased brain atrophy and higher disability over time in adults (Reiss et al., 2023). The IgG index is another CSF measure that may be helpful in the evaluation of inflammatory brain disease. IgG is typically produced peripherally however in specific neuroinflammatory conditions, such as multiple sclerosis, it is produced within the central nervous system (CNS) (Joubert and Dalmau, 2019). Intrathecal IgG production has been reported to be elevated in both pediatric and adult multiple sclerosis, although the most sensitive marker remains the presence of oligoclonal bands which have been reported to be found in as many as 98% of patients with early-onset multiple sclerosis and 96% of adults with multiple sclerosis (Pohl et al., 2004; Link and Huang, 2006; Yu et al., 2020). Although oligoclonal bands are a more sensitive marker for multiple sclerosis, evaluation of the IgG index may be a diagnostically valuable tool in synonymous neurologic conditions.

Pediatric acute-onset neuropsychiatric syndrome (PANS) is a neuropsychiatric condition characterized by abrupt-onset of obsessive-compulsive symptoms and/or eating restriction and with at least two additional acute onset neuropsychiatric changes (Swedo et al., 2012; Chang et al., 2015). Imaging studies suggest involvement of basal ganglia (Giedd et al., 2000; Kumar et al., 2015; Cabrera et al., 2019; Zheng J. et al., 2020) including diffuse microstructural brain changes that prominently affect the basal ganglia and other regions of the brain (Kadry et al., 2020). In 2012 we created the Immune Behavioral Health (IBH) clinic to serve patients with PANS and further clinical research of this condition. Patients are pre-screened prior to clinic entry to increase the probability that their case fits the definition of PANS. Nonetheless, approximately 50% of the patients coming to clinic do not meet PANS diagnostic criteria; these patients were included in this report since they are an important and highly impaired patient group who deserve a medical evaluation despite not meeting criteria for PANS or AE and because this subgroup has been found to have low-level CSF abnormalities and/or other inflammatory conditions including arthritis (Ma, 2024). There are no published studies examining CSF changes in children diagnosed with PANS.

Here, we report the psychiatric symptoms and CSF findings of consecutive pediatric patients undergoing lumbar puncture for the evaluation of severe (acute and sub-acute) psychiatric deteriorations.

## Methods

This retrospective cohort study was approved by the Stanford University Institutional Review Board (retrospective IRB = 28533, and prospective IRB = 26922).

We reviewed the records of 471 consecutive subjects evaluated between September 15, 2012 and May 13, 2023 for psychiatric/behavioral deteriorations. Electronic medical records were systematically searched using a navigational search method. CSF data was collected from the electronic medical record (from both the primary study institution and outside facilities) and entered our IRB-approved REDcap (Harris et al., 2009, 2019) database.

Inclusion criteria for this study required that subjects presented with neuropsychiatric deterioration at time of first clinic visit and had had a lumbar puncture completed as part of their diagnostic evaluation. Subjects with lumbar punctures performed at other time points (i.e., neonatal sepsis) for other reasons were excluded. If multiple lumbar punctures were performed, only the first lumbar puncture was included unless a particular parameter was missing; in this case, missing data would be collected from the subsequent lumbar puncture. The final study cohort included 71 subjects who had undergone lumbar puncture upon initial presentation of the neuropsychiatric deterioration.

Subjects were categorized into three categories based on the following groups: PANS, autoimmune encephalitis (AE), and “other neuropsychiatric deterioration” not classifiable as PANS or AE. Patients were classified as PANS based on published criteria (Swedo et al., 2012; Chang et al., 2015) by a psychiatrist (MT, MS, XJ). Patients were classified as having autoimmune encephalitis based on published (provisional) pediatric AE classification criteria (Cellucci et al., 2020). Patients not meeting criteria for PANS or AE were assigned to the “other neuropsychiatric deterioration” group which was a mix of patients with sub-acute onset of severe psychiatric symptoms spanning multiple domains including OCD, eating/fluid restriction, behavioral regression, psychosis, etc. with suspected immunopsychiatric condition. For patients diagnosed with PANS, acuity is included as part of the diagnostic criteria.

Demographic and clinical data including age at presentation, race, ethnicity, biological sex, and presenting psychiatric symptoms were collected. CSF measures include CSF cell counts, total protein, albumin quotient (Qalb), IgG index and the presence of oligoclonal bands. To assess whether these values were abnormally elevated, we used age-specific reference values for CSF total protein and the albumin quotient and non-age dependent cut-off values for the IgG Index (Pohl et al., 2004; Kahlmann et al., 2017; Zheng Y. et al., 2020). Leukocyte counts were excluded if patient had a traumatic tap (CSF red blood cell count > 200) since peripheral cell count data was not available for all subjects to calculate correction for number of red blood cells present (Mehl, 1986).

Clinical data indicative of systemic autoimmune mediated processes, immune dysregulation, endothelial changes (vasculopathy or vasculitis), and arthritis were also collected and categorized by type of neuropsychiatric deterioration. Autoimmune markers included antinuclear, anti-histone, and anti-thyroid antibodies, c1q binding assay, and c3 and c4 complements. Immune dysregulation features include presence of leukopenia, thrombocytosis, elevated c-reactive protein and elevated erythrocyte sedimentation rate. Laboratory and clinical markers of vasculopathy and vasculitis were also collected including von Willebrand factor, D-dimer, and clinical presence of onychodermal bands, livedo reticularis, periungual redness and palatal petechiae. Data regarding presence of arthritis and arthritis subtype (both at initial visit and in subsequent visits) is reported. Psychiatric/behavioral symptoms at time of first clinical visits were initially evaluated as a composite of clinical data and symptom inventory checklist by psychiatry team members MS, YX, and MT. This data was extracted and the number of subjects with a range of symptoms were reported by diagnostic subgroups: PANS, “other neuropsychiatric deterioration” (heterogeneic psychiatric diagnoses) and AE.

## Results

In total, records of 471 patients in the IBH clinic were reviewed and of these patients, 71 patients underwent lumbar puncture. Lumbar puncture is not a routine part of evaluation for PANS, and patients underwent LP for multiple reasons as part of diagnostic evaluation for other neurologic disease. The mean age at clinic presentation for those who underwent lumbar puncture was 12.3 years (Table 1). Most patients seen in the IBH clinic identified with white or Asian race and the majority of patients identified as non-Hispanic.

**TABLE 1:** Characteristics of subjects evaluated in the Stanford Immune Behavioral Health Clinic on presentation.

	No LP (*n* = 398)	LP (*n* = 71)
**Age at clinic presentation in years**		
Mean (SD)	10.8 (4.5)	12.3 (3.8)
Range (median)	2.5–48.0 (10.5)	4.1–22.4 (12.4)
**Sex, n (%)**		
Male	222 (56)	43 (61)
Female	176 (44)	28 (39)
**Race, n (%)**		
White	316 (79)	53 (75)
Multiracial	55 (14)	12 (17)
Asian	20 (5)	5 (7)
Unknown	4 (1)	1(1)
**Ethnicity, n (%)**		
non-Hispanic	356 (89)	57 (80)
Hispanic	42 (11)	14 (20)

Of the 71 subjects who met inclusion criteria (had lumbar punctures), 35 were classified as PANS, 31 were classified as “other neuropsychiatric deterioration” and 5 were classified as AE (Table 2) based on a pediatric autoimmune encephalitis algorithm (Cellucci et al., 2020). With respect to psychiatric and behavioral symptoms at presentation, the most common symptoms in the PANS cohort were obsessive compulsive behaviors and anxiety. The most common symptoms in the “other neuropsychiatric deterioration” group were emotional lability/mood swings, obsessive compulsive behaviors, and anxiety (Table 2); about half of this subgroup (*n* = 15) would have met diagnostic criteria for PANS except that their clinical presentation was subacute. All patients with autoimmune encephalitis had emotional lability/mood swings. In contrast to the PANS group, relatively few patients in the autoimmune encephalitis group had obsessive compulsive behaviors and behavioral regression.

**TABLE 2:** Neuropsychiatric symptoms of 71 patients presenting for PANS evaluation who underwent LP.

Psych/behavioral symptoms at first clinic presentation for those who underwent LP	PANS (*n* = 35), *n* (%)	Other neuropsych det^&^ (*n* = 31), *n* (%)	AE* (*n* = 5), *n* (%)
Obsessions Compulsions Hoarding	31 (89)	22 (71)	1 (20)
Food refusal Fluid refusal	21 (60)	15 (48)	1 (20)
Separation anxiety Other anxiety/fears/phobias/panic attacks	30 (86)	22 (71)	3 (60)
Mood swings/moodiness Emotional lability (inappropriate crying or laughing spells)	26 (74)	23 (74)	5 (100)
Suicidal ideation/behavior	9 (26)	7 (23)	3 (60)
Depression/sadness	20 (57)	15 (48)	4 (80)
Irritability	26 (74)	19 (61)	4 (80)
Aggressive behaviors violence, and/or rage	22 (63)	21 (68)	4 (80)
Hyperactivity or impulsivity Inattention	26 (74)	19 (61)	3 (60)
Baby talk Other behavioral/developmental regression	6 (17)	15 (48)	4 (80)
Handwriting deterioration	12 (34)	10 (32)	2 (40)
Cognition	22 (63)	19 (61)	3 (60)
Pain (headaches, abdominal pain, body pain)	18 (51)	13 (42)	4 (80)
Sleep disturbance	22 (63)	19 (61)	4 (80)
Enuresis Urinary frequency	16 (46)	7 (23)	1 (20)
Sensory issues/amplification	20 (57)	17 (55)	3 (60)
Hallucinations Delusions or paranoid thoughts	11(31)	8 (26)	3 (60)
Motor tics Phonic tics	17 (49)	12 (39)	3 (60)

*Includes Steroid Responsive Encephalopathy Associated with Thyroid Antibodies (SREAT) (*n* = 1), voltage gated potassium channel (VGKC) encephalitis (*n* = 1), seronegative encephalitis (*n* = 2), and Rasmussen’s (*n* = l). ^&^Other Neuropsychiatric Deterioration: Patients presenting for IBH/PANS eval who did not meet strict criteria for PANS. Differences may include sub-acute onset of OCD and other psychiatric symptoms.

CSF indices including CSF white blood cell count (WBC), protein, albumin quotient, and IgG index are reported in Table 3. CSF abnormalities were found in all three diagnostic subgroups. Elevated CSF protein was seen in 40% of patients in the autoimmune encephalitis group, 26% of patients in the PANS group, and 37% of patients in the “other neuropsychiatric disease” category. The mean albumin quotient was highest in the “other neuropsychiatric” group (4.5 x 10^–3^) although those with autoimmune encephalitis had a similar proportion of subjects with abnormally elevated levels (25%). Approximately 9% of those with PANS had an abnormally elevated albumin quotient. Few subjects had abnormal IgG index values: PANS group (*n* = 1), “other neuropsychiatric deterioration” (*n* = 2), autoimmune encephalitis (*n* = 0). Oligoclonal bands were present in three patients (one in each group). Pleocytosis was not observed in PANS or the “other” group. In total, CSF abnormalities were found in 40% of patients with autoimmune encephalitis, 45% of the “other neuropsychiatric disorders”, and 29% of patients with PANS.

**TABLE 3:** CSF analysis of 71 patients presenting for PANS evaluation who underwent LP.

	PANS (*n* = 35)	Other neuropsych det^&^ (*n* = 31)	AE^Δ^ (*n* = 5)
**Age at LP in years**			
Mean (±SD)	11 (±5.0)	12.8 (3.2)	12 (±3.3)
Median (range)	11 (4–22)	13.6 (5.6–19.2)	12 (8–16)
**White blood cell count (WBC)** ^Δ^	*n* = 33	*n* = 26	*n* = 5
<1 WBC present, *n*	5	9	0
Mean WBCs present	1.3	0.8	3.8
Median (range)	1 (0–3)	1 (0–3)	1 (1–15)
**CSF total protein (mg/dL)**	*n* = 34	*n* = 30	*n* = 5
Mean (± SD)	27 (±11)	30 (±11)	30 (±16)
Median (range)	23	29	27
n elevated for age group (%)*	9/34 (26)	11/30 (37)	2/5 (40)
**Qalb (1×10−3)**	*n* = 22	*n* = 20	*n* = 4
Mean (±SD)	3.5 (±2.2)	4.5 (2.0)	3.4 (±1.5)
Median (range)	2.8 (1.3–11)	3.8 (1.6–8.3)	3.1 (2.0–5.4)
n elevated for age group (%)**	2/22 (9.1)	5/20 (25)	1/4 (25)
**lgG Index**	*n* = 22	*n* = 20	*n* = 4
Mean (±SD)	0.56 (±0.19)	0.55 (±0.19)	0.46 (±0.08)
Median (Range)	0.51 (0.41–1.3)	0.49 (0.17–1.17)	0.43 (0.41–0.58)
n elevated (%)***	1/22 (4.5)	2/20 (10)	0/4
**Oligoclonal bands**	*n* = 24	*n* = 19	*n* = 1
n present (%)	1/24 (4.2)	1/19 (5.3)	1/4 (25)
# of bands present	>4	>4	1
*1 or more CSF parameters out of range n (%)^Ψ^*	10/35 (29)	14/31(45)	2/5 (40)

Includes Steroid Responsive Encephalopathy Associated with Thyroid Antibodies (SREAT), voltage gated potassium channel (VGKC) encephalitis, and seronegative encephalitis. ^&^Other Neuropsychiatric Deterioration: Patients presenting for IBH/PANS eval who did not meet strict criteria for PANS. Differences may include sub- acute onset of OCD and other psychiatric symptoms. ^Δ^Excluded patients with traumatic taps (Mehl, 1986) where CSF red blood cell count > 200. *Cut-off values for CSF Total Protein were 25, 28, 34, and 45 mg/dL for the age categories of 2–6, 6–12, 12–18, and 18–25 years old, respectively (Kahlmann et al., 2017). **Cut-off value for albumin quotient (QAlb) used for children < 16 years old is 5×10−3 and 7×10−3 for those >16 years old (Pohl et al., 2004). ***Cut-off value for IgG Index was 0.70 (Zheng Y. et al., 2020). ^Ψ^Includes parameters for CSF total protein, Qalb, IgG Index and oligoloclonal bands.

Of the three patients who had oligoclonal bands, the first patient met classification criteria for PANS. This first case was highly unusual because he had persistent psychotic symptoms which is atypical in PANS, where psychosis tends to be transient (Silverman et al., 2019). The second patient had a subacute deterioration and did not meet criteria for PANS nor AE. By the time this patient arrived to the IBH clinic, he had a relapsing-remitting course and presented with catatonia and elevated ASO. Upon warming his trunk, an erythema marginatum rash emerged. Although he had no signs of chorea while catatonic, we suspected that his condition was related to Sydenham chorea/acute rheumatic fever (ARF) given the classic rash of ARF (erythema marginatum). The last patient with oligoclonal bands had multiple abnormal CSF findings (including pleocytosis) and was diagnosed with AE. The patient presented with acute-onset confusion/combativeness along with other neuropsychiatric changes and was diagnosed with and treated for seronegative autoimmune encephalitis based on clinical features and CSF findings including elevated protein and presence of oligoclonal bands.

Three patients had an elevated IgG index: The first patient met PANS criteria, had preceding group A streptococcal infection, and presented with arthritis at initial PANS deterioration (Ma et al., 2023). Interestingly, this patient also had elevated antibodies to thyroid peroxidase (TPO), elevated anti-histone antibody, low memory B cells, high immune complexes (C1 q binding assay). The second patient had a sub-acute presentation of severe OCD and suicidality coincident with Hashimoto’s thyroiditis but did not meet criteria for AE. The last patient had autism and presented with subacute-onset onset of severe OCD, random irregular movements of the face (subtle chorea), disabling rituals, severe tics, aggression, and loss of self-care. At time of presentation, the patient had a throat swab (PCR positive for group A strep), synovitis of bilateral first metatarsal phalangeal joints, prominent onychodermal bands, anemia, and a first-degree family member with suspected acute rheumatic fever.

With respect to other systemic features, Table 4 summarizes the presence of autoimmune markers and clinical features, markers of immune dysregulation, and vasculopathy/vasculitis by diagnosis. Trends include higher proportions of patients in the PANS group with the presence of onychodermal bands and livedo reticularis. Considering abnormal markers of immune dysregulation, only one patient from each subgroup had an elevated ESR and no patients in the “other neuropsychiatric deterioration” or the autoimmune encephalitis groups had other abnormal markers. Other autoantibodies including anti-nuclear antibody (ANA) and anti-histone antibodies, were seen in both the PANS and “other neuropsychiatric deterioration groups,” but only one patient with autoimmune encephalitis had anti-thyroid antibodies. Arthritis was seen in both the PANS and “other” groups, but not seen in the AE group. The most common subtype of arthritis amongst the PANS group was post-infectious/transient arthritis. The most common subtype of arthritis seen in the “other neuropsychiatric deteriorations” group was enthesitis related arthritis (ERA) as per International League of Associations for Rheumatology (ILAR) criteria.

**TABLE 4:** Markers and clinical features of autoimmunity, immune dysregulation and vasculopathy/vasculitis in 71 patients presenting for PANS evaluation who underwent LP.

	PANS (*n* = 35)	Other neuropsych det^&^ (*n* = 31)	AE* (*n* = 5)
**Autoimmune markers (non-specific), *n***			
Antinuclear antibody (ANA), *titer ≥ 1:80*	3/30	2/29	0
Anti-histone antibody, *high*	1/31	4/29	0
Anti-thyroid antibodies, *high*	7/31	6/26	1/5
C1q binding assay, *high*	5/30	6/27	0
Complement 3, *low*	5/31	1/27	0
Complement 4, *low*	8/31	6/27	0
**Immune dysregulation/inflammation markers (non-specific), *n***			
Leukopenia, *white blood cell < 4,000/mm^3^*	2/31	0/27	0/5
Thrombocytosis, *platelet* >*400,000/mm^3^*	2/31	0/27	0/5
C-reactive protein (CRP), *high*	1/31	0/27	0/5
Erythrocyte sedimentation rate (ESR), *high*	1/31	1/27	1/5
**Vasculopathy/vascular inflammation markers (non-specific), *n***			
von Willebrand factor antigen, *high*	2/31	3/27	2/5
D-dimer, *high*	1/31	2/27	1/4
Onychodermal band, *abnormally prominent*	10/35	6/31	1/5
Livedo reticularis, *present*	11/35	3/31	0/5
Periungual redness and swelling, *present*	3/35	1/31	0/5
Palatal petechiae, *present*	2/35	2/31	0/5
**Arthritis**			
Individuals with arthritis, *n*	9/35	7/31	0/5
ILAR enthesitis related arthritis (ERA)	2	4	
ILAR pesoriatic arthritis	2	2	
ILAR persistant oligoarthritis	1	1	
Post-infectious Arthritis/Transient Arthritis	3	1	

*Includes Steroid Responsive Encephalopathy Associated with Thyroid Antibodies (SREAT) (*n* = 1), voltage gated potassium channel (VGKC) encephalitis (*n* = 1), seronegative encephalitis (*n* = 2), and Rasmussen’s (*n* = 1). ^&^Other Neuropsychiatric Deterioration: Patients presenting for IBH/PANS eval who did not meet strict criteria for PANS. Differences may include sub-acute onset of OCD and other psychiatric symptoms.

## Discussion

This is the first study to describe clinical CSF findings in a cohort of patients classified as PANS who underwent lumbar puncture for CSF evaluation. The minority of patients presenting to our IBH clinic underwent LP (71/471) and thus this patient group is biased toward having an unusually severe presentation and/or had atypical features for PANS and/or they were in their initial deterioration and per the pediatric AE classification criteria algorithm underwent the recommended full AE evaluation (Cellucci et al., 2020).

PANS is primarily a relapsing/remitting disorder and most patients come to the attention of subspecialty clinics with a history of prior deteriorations that self-resolve or resolve with minimal interventions, including treatment/resolution of infection, NSAIDS, or a brief steroid course. In most cases, this classical presentation does not warrant a LP. However, at the outset of the initial neuropsychiatric episode, symptoms alone may not be distinguishable between PANS, autoimmune encephalitis, and other inflammatory brain disorders, and thus EEG, MRI, or LP may be indicated (Chang et al., 2015; Cellucci et al., 2020). In new-onset cases without the history of prior deteriorations, clinicians must consider obtaining a LP if indicated by the provisional pediatric autoimmune encephalitis classification criteria (Cellucci et al., 2020) or due to other symptoms that suggest an inflammatory brain disorder for which LP is indicated.

Among the patients who met strict criteria for PANS and underwent LP, we found that approximately one-quarter had at least one CSF abnormality. Pleocytosis was not observed in the PANS group, but remain critical in ruling out infection in the setting of abrupt mental status changes. The most frequently seen CSF abnormalities were elevated CSF total protein (26%) and elevated albumin quotient (9%). In addition to these subtle CSF abnormalities, these patients also had features of inflammation/autoimmunity including low complements and/or high immune-complexes, non-specific autoantibodies, vasculopathy signs (skin and labs), and arthritis (primarily transient arthritis, post-infectious arthritis or enthesitis related arthritis).

An unexpected result was the higher frequency of CSF abnormalities (45%) in the “other psychiatric deterioration” group. This group is comprised of patients who were referred to our clinic for a PANS evaluation (driven by either the parent or pediatrician) but who did not meet the strict PANS criteria (i.e., acute-onset) nor the AE criteria (Cellucci et al., 2020). The significance of these differences in CSF abnormalities unclear, but may reflect longer duration of symptoms prior to LP (due to the subacute nature of the presentation) and/or higher rate of co-existing autoimmune/inflammatory conditions. Three patients in this group had significant CSF abnormalities: IgG bands (*n* = 1) and elevated IgG index (*n* = 2) and also had co-existing inflammatory conditions (thyroiditis, arthritis, and/or suspicion of Sydenham chorea). Similar to patients in the PANS cohort, this group also had clinical features suggestive of systemic inflammation/autoimmunity including low complements and/or high immune-complexes, non-specific autoantibodies, vasculopathy signs (skin and labs), and arthritis. Most of the patients in the “other”/sub-acute group had obsessive compulsive behaviors (71%) and food/fluid restrictions (48%) similar to the PANS group (Table 2).

The most notable feature of both the PANS and “other”/subacute groups was the high frequency of arthritis which was previously reported to affect 1/3 of the PANS group and 1/3 of the non-PANS/non-AE group (Ma, 2024). In the PANS cohort, the neuropsychiatric symptoms typically predate the presentation of arthritis (Ma, 2024) but this has not been fully examined in the “other neuropsychiatric” group. The commonalities between the acute and “other neuropsychiatric deterioration” groups suggests overlap between these groups. The arthritis and vasculopathy features highlights the systemic (albeit subtle) nature of the inflammation with possible impact at the BBB (given that 1/3 have elevated protein and/or albumin quotient). The connection between OCD and eating disorders with autoimmunity has been established by large and rigorous epidemiological studies (Zerwas et al., 2017; Gromark et al., 2019).

Our study was not designed nor powered to determine the significance of the differences between these convenience subgroups. This descriptive study, on the other hand, may suggest overlap in this group with regards to the psychiatric symptoms and inflammation features which may involve complement activation and low grade vascular inflammation (Ma, 2024) as is recently been reported in Long Covid (Cervia-Hasler et al., 2024; Forte, 2024).

Although autoantibodies have been described in many autoimmune/inflammatory diseases, it is well known that autoantibodies (ANA, anti-thyroid, anti-NMDA, etc.) are common in the healthy population and alone are not sufficient to cause disease (Sakata et al., 1994; Steiner et al., 2014; Dinse et al., 2020) despite their potential role and association with disease. A number of autoantibodies have been described in PANS, Pediatric Autoimmune Neuropsychiatric Disorder Associated with Streptococcal infection (PANDAS), and Sydenham chorea (Morris et al., 2009; Cox et al., 2015; Hesselmark and Bejerot, 2017; Frye and Shimasaki, 2019; Shimasaki et al., 2020). However, as in other inflammatory diseases, there are challenges with regards to sensitivity and specificity of these presumed autoantibodies. The most recently discovered and rigorously studied autoantibodies in PANS/PANDAS target cholinergic interneurons in the basal ganglia and have been shown to be present in higher titers in patients compared to controls (Frick et al., 2018; Xu et al., 2021). These anti-cholinergic interneuron autoantibodies have been shown to have metabolic and electrophysiologic impact on these regulating interneurons; the reduced function in turn disrupts their role in regulating behavior, learning, mood, and movement (the cardinal symptoms of PANS and Sydenham’s chorea) (Kirvan et al., 2003, 2006; Frick et al., 2018). Although these studies suggest a role for autoantibodies, it is clear that healthy controls may have a low level of these antibodies. Thus, disease factors in addition to autoantibodies are likely playing a role. Our findings of elevated CSF protein in about a quarter of patients with PANS and elevated CSF protein and albumin quotient in patients with “other neuropsychiatric deterioration” may indicate a partial disruption in BBB, which may allow access of these autoantibodies to relevant brain structures (basal ganglia). Our preliminary findings suggest the need for research into the effects of PANS plasma (and plasma from related conditions) on BBB models to investigate the relationship between BBB disruption and disease state (i.e., does worse BBB impairment correspond to worse psychiatric symptoms?).

Although elevated CSF protein and albumin quotient were relatively common in this patient population, other measures of CNS autoimmunity were rare (elevated IgG index, presence of oligoclonal bands). The findings of elevated CSF protein and albumin quotient may reflect worse neuropsychiatric severity given that patients had undergone LP based on being unusually severe or atypical of PANS, thus these cases may be more closely related to other diagnostic categories (AE, Sydenham chorea, neuropsychiatric lupus, etc.).

Patients with AE typically have subacute-onset of cognitive and memory difficulties, often followed by seizures or coma. Psychiatric symptoms are common early in the course of AE and may include psychosis, compulsive behaviors, panic attacks, and other behavior changes (aggression, inappropriate sexual behaviors, etc.) (Lancaster, 2016). For the subjects within the PANS cohort, psychiatric symptoms were acute or hyperacute in onset, disease course was relapsing and remitting, and OCD was the predominant phenotype, as expected given that these patients met PANS classification criteria (Chang et al., 2015). In review of clinical features of those in the “other neuropsychiatric deterioration” group, approximately half met PANS criteria with the exception that their onset was subacute. Although this study was not designed to specifically compare the behavioral characteristics between groups, it is notable that OCD was less common among the 4 patients meeting criteria for AE.

There are several limitations in our study. First, the subgroup of patients who underwent LP is presumably a unique subgroup which biases the results. Most patients presenting to the IBH clinic did not undergo LP (398/471, 84.5%) and the patients who underwent LP likely had more severe presentation necessitating LP as part of their diagnostic workup. Since patients are admitted to clinic based on a high likelihood of meeting PANS criteria, the number of patients from the non-PANS subgroups were overall low and as such we were not able to evaluate for statistically meaningful differences between the 3 diagnostic subgroups.

This study characterizes the CSF protein characteristics observed in children with severe neuropsychiatric deteriorations and corresponding clinical features. By beginning to connect CSF features in these patients, we are taking a needed first step toward investigating what happens at the level of the blood brain barrier. Further work is required to elucidate the mechanisms that link post-infectious inflammatory processes and autoimmunity with psychiatric symptoms.

## Data availability statement

The raw data supporting the conclusions of this article will be made available by the authors, without undue reservation.

## Ethics statement

The studies involving humans were approved by the Stanford University Institutional Review Board. The studies were conducted in accordance with the local legislation and institutional requirements. Written informed consent for participation in this study was provided by the participants’ legal guardians/next of kin.

## Author contributions

RP: Conceptualization, Formal analysis, Investigation, Methodology, Writing – original draft, Writing – review and editing. WZ: Conceptualization, Data curation, Formal analysis, Investigation, Methodology, Project administration, Writing – original draft, Writing – review and editing. MM: Data curation, Formal analysis, Investigation, Writing – review and editing. MS: Investigation, Writing – review and editing. YX: Investigation, Writing – review and editing. BF: Writing – review and editing. MT: Writing – review and editing. EM: Writing – review and editing. JF: Conceptualization, Formal analysis, Funding acquisition, Investigation, Methodology, Project administration, Resources, Supervision, Writing – original draft, Writing – review and editing.
